# mTOR drives cerebrovascular, synaptic, and cognitive dysfunction in normative aging

**DOI:** 10.1111/acel.13057

**Published:** 2019-11-06

**Authors:** Candice E. Van Skike, Ai‐Ling Lin, Raquel Roberts Burbank, Jonathan J. Halloran, Stephen F. Hernandez, James Cuvillier, Vanessa Y. Soto, Stacy A. Hussong, Jordan B. Jahrling, Martin A. Javors, Matthew J. Hart, Kathleen E. Fischer, Steven N. Austad, Veronica Galvan

**Affiliations:** ^1^ Department of Cellular and Integrative Physiology Barshop Institute for Longevity and Aging Studies University of Texas Health San Antonio San Antonio Texas; ^2^ The Glenn Biggs Institute for Alzheimer's & Neurodegenerative Diseases University of Texas Health San Antonio San Antonio Texas; ^3^ Sanders‐Brown Center on Aging Department of Pharmacology and Nutritional Sciences Department of Biomedical Engineering Department of Neuroscience University of Kentucky Lexington Kentucky; ^4^ Department of Veterans Affairs South Texas Veterans Health Care System San Antonio Texas; ^5^ Department of Psychiatry University of Texas Health San Antonio San Antonio Texas; ^6^ Center for Innovation in Drug Discovery Cancer Therapy and Research Center, and the Department of Biochemistry University of Texas Health San Antonio San Antonio Texas; ^7^ RNAi/CRISPR High Throughput Screening Facility Greehey Children's Cancer Research Institute University of Texas Health San Antonio San Antonio Texas; ^8^ Department of Biology and Nathan Shock Center of Excellence in the Basic Biology of Aging University of Alabama at Birmingham Birmingham Alabama

**Keywords:** aging, brain vasculature, cerebral blood flow, cognitive decline, functional MRI, mTOR

## Abstract

Cerebrovascular dysfunction and cognitive decline are highly prevalent in aging, but the mechanisms underlying these impairments are unclear. Cerebral blood flow decreases with aging and is one of the earliest events in the pathogenesis of Alzheimer's disease (AD). We have previously shown that the mechanistic/mammalian target of rapamycin (mTOR) drives disease progression in mouse models of AD and in models of cognitive impairment associated with atherosclerosis, closely recapitulating vascular cognitive impairment. In the present studies, we sought to determine whether mTOR plays a role in cerebrovascular dysfunction and cognitive decline during normative aging in rats. Using behavioral tools and MRI‐based functional imaging, together with biochemical and immunohistochemical approaches, we demonstrate that chronic mTOR attenuation with rapamycin ameliorates deficits in learning and memory, prevents neurovascular uncoupling, and restores cerebral perfusion in aged rats. Additionally, morphometric and biochemical analyses of hippocampus and cortex revealed that mTOR drives age‐related declines in synaptic and vascular density during aging. These data indicate that in addition to mediating AD‐like cognitive and cerebrovascular deficits in models of AD and atherosclerosis, mTOR drives cerebrovascular, neuronal, and cognitive deficits associated with normative aging. Thus, inhibitors of mTOR may have potential to treat age‐related cerebrovascular dysfunction and cognitive decline. Since treatment of age‐related cerebrovascular dysfunction in older adults is expected to prevent further deterioration of cerebral perfusion, recently identified as a biomarker for the very early (preclinical) stages of AD, mTOR attenuation may potentially block the initiation and progression of AD.

## Graphical abstract

Aging is associated with increased cerebrovascular dysfunction, synaptic loss, and cognitive decline. Attenuation of mTOR negates these age‐associated changes in rats, suggesting that mTOR activity underlies the etiology of age‐associated microvascular and neuronal dysfunction and may thus drive cognitive impairment in normative aging.

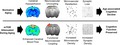

## 1 INTRODUCTION

Normal brain aging predisposes vulnerable neurons to degeneration and is associated with cognitive decline and an increased likelihood of developing a neurodegenerative disorder (Mattson & Magnus, 2006). The prevalence of Alzheimer's disease (AD), the most common cause of dementia in the elderly, is expected to double approximately every 20 years, with 131.5 million cases expected worldwide by 2050 (Prince et al., 2015). The prevalence of AD is increasing rapidly, yet there is no disease‐modifying treatment currently available. Although age is a primary risk factor for AD, very little is known about the molecular mechanisms that link the regulation of brain aging to neurodegenerative diseases of advanced age.

Cerebrovascular dysfunction is a universal feature of aging (Zlokovic, 2011) that includes impaired endothelium‐dependent vasodilation and global and regional decreases in cerebral blood flow (CBF) (Martin, Friston, Colebatch, & Frackowiak, 1991). While decreased CBF does not indicate a particular disease state, reduced CBF is associated with impaired cognitive function (Wang et al., 2016) and decreased neuronal plasticity (Hamadate et al., 2011). Using functional magnetic resonance imaging (fMRI), which measures changes in CBF to infer neuronal activation, it is apparent that the human brain can reorganize and redistribute functional networks to compensate for nonpathologic age‐related impairments. In general, task‐related neural activation becomes more diffuse with advancing age as other brain regions are recruited to maintain task proficiency. However, when cognitive demand exceeds these compensatory mechanisms, performance becomes impaired (Cabeza et al., 1997). Because cerebrovascular dysfunction plays a critical role in the pathogenesis of age‐related neurodegenerative disorders, including AD (Csiszar et al., 2017; Lin et al., 2017, 2013; Van Skike et al., 2018), and manifests early in disease progression (Iturria‐Medina, Sotero, Toussaint, Mateos‐Perez, & Evans, 2016), it is important to investigate the mechanisms underlying cognitive decline and brain vascular deterioration driven by nonpathologic aging.

The mammalian/mechanistic target of rapamycin (mTOR) pathway regulates aging in mammals (Wilkinson et al., 2012). mTOR is also expressed throughout the brain, where it is linked to synaptic plasticity and learning and memory (Tang et al., 2002) through the regulation of protein synthesis (Tang et al., 2002) and autophagy (Mizushima, Levine, Cuervo, & Klionsky, 2008). In the aging brain, autophagy is reduced, contributing to the accumulation of aggregated proteins and neurodegeneration (Komatsu et al., 2006). Thus, dysregulation of the mTOR pathway during nonpathologic aging may contribute to cognitive decline, cerebrovascular dysfunction, and a predisposition toward developing neurodegenerative diseases associated with advanced age.

We have previously shown that mTOR inhibition attenuates cognitive dysfunction in aged mice (Halloran et al., 2012). We and others have also shown that chronic mTOR attenuation with rapamycin can prevent and reverse cognitive and cerebrovascular deficits in several independent mouse models of AD (Lin et al., 2017, 2013;Van Skike et al., 2018) and vascular cognitive impairment (Jahrling et al., 2018), leading to improved cerebrovascular function and preserved cognitive outcomes in these models of age‐related disease. The contribution of mTOR to cerebrovascular deficits is associated with normative aging, though its impact on cognitive outcomes remains unknown. The goal of this study was to test the hypothesis that mTOR drives cerebrovascular and synaptic dysfunction during aging and that chronic mTOR inhibition with rapamycin mitigates nonpathologic age‐related deterioration of cognition and cerebrovascular function in aged rats without underlying disease.

## 2 RESULTS

### 2.1 Age‐related decline in hippocampal‐dependent learning and memory is driven by mTOR

**Figure 1:**
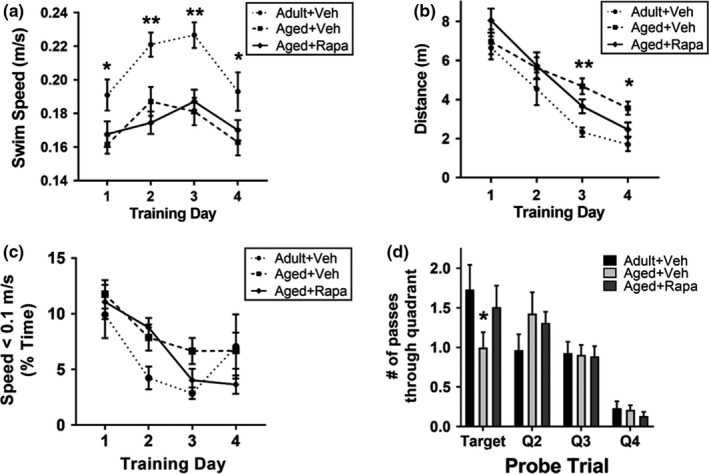
Age‐associated cognitive decline in rats is driven by mTOR. (a) Thirty‐four‐month old aged rats, regardless of treatment condition, displayed slower swim speed compared with 16‐month‐old adults (for each training day, q(12)<4.60, *p < .018; ** indicates *p* < .01). Tukey's *post hoc* tests were applied to a significant main effect of group, *F*(2,36)=8.96, *p* = .0007 in two‐way repeated measures ANOVA analyses. (b) Aged rats exhibit spatial learning and memory impairments compared with adults (*F*(2, 36)=5.40, *p* = .009), especially on days 3 (**q(144)=4.23, *p* = .009) and 4 (*q(144)=3.35, *p* = .049) of training in the Morris water maze (MWM). Performance of aged rats in which mTOR was chronically attenuated with rapamycin (aged + rapa) did not significantly differ from that of adult rats for each training day (q(144)<2.56, *p* < .17, n.s.). Tukey's *post hoc* tests were applied to significant main effects of day (*F*(3,108) = 48.59, *p* < .0001) and group (*F*(2,36)=5.40, *p* = .009) in two‐way ANOVA (day x group) with repeated measures analyses. (c) The proportion of swimming slower than 0.10 m/s decreases with training (*F*(3,108)=12.87, *p* < .0001), but is not different among groups (*F*(2, 36)=1.93, *p* = .16). (d) Aged rats exhibit significant spatial memory impairment in the probe trial compared with adult rats (*q(144)=3.81, *p* = .021). Inhibition of mTOR restores spatial memory in aged rats (aged + rapa group) to a level indistinguishable from that of adult animals (q(144)=1.17, *p* = .69). Tukey's post hoc tests were applied to a significant main effect of group (*F*(3, 108)=24.86 *p* < .0001) via two‐way ANOVA. Data are presented as mean ± *SEM* (*n* = 10‐15/group)

To determine the contribution of mTOR to cognitive deficits during normative aging, we used the Morris water maze to measure hippocampal‐dependent spatial learning and memory in adult (16 months old) and aged (34 months old) rats that were either fed either a diet with empty microcapsules or a diet containing microencapsulated rapamycin at 14 parts per million (ppm) for 15 months starting at 19 months of age. Consistent with prior studies (Novier, Van Skike, Diaz‐Granados, Mittleman, & Matthews, 2013), we found that adult rats swam significantly faster than aged rats, regardless of treatment condition (Figure 1a). Thus, we used path length as measure of performance during training in the MWM since this measure is not impacted by swim speed. Total distance swam declined progressively throughout the 4 days of training (Figure 1b), indicating effective spatial learning among the groups. However, 34‐month‐old aged rats had significantly longer path lengths than the 16‐month‐old adult rats on training days 3 and 4. No significant differences in path length were observed in aged rats treated with rapamycin as compared to adult rats on any training day. Additionally, there were no differences among the groups in the amount of slow swimming below 0.10 m/s during acquisition (Figure 1c).

During a 24‐hr probe trial, aged rats made significantly fewer passes over the learned location of the hidden platform as compared to adult rats during a 24‐hr recall probe trial (Figure 1d). Recall of the hidden platform location in aged rats treated with rapamycin, however, was indistinguishable from that of adult rats. Together, these data indicate that deficits in spatial learning and memory in aged rats can be negated by mTOR attenuation, suggesting that spatial learning and memory impairments in aged rats are at least partially driven by mTOR. Of note, chronic mTOR inhibition did not rescue age‐related decreases in swim speed, ruling out an impact of mTOR attenuation on neuromotor pathways or muscle function and activity required for swimming.

### 2.2 mTOR drives sensory‐evoked functional hyperemia impairments in rats of advanced age

**Figure 2:**
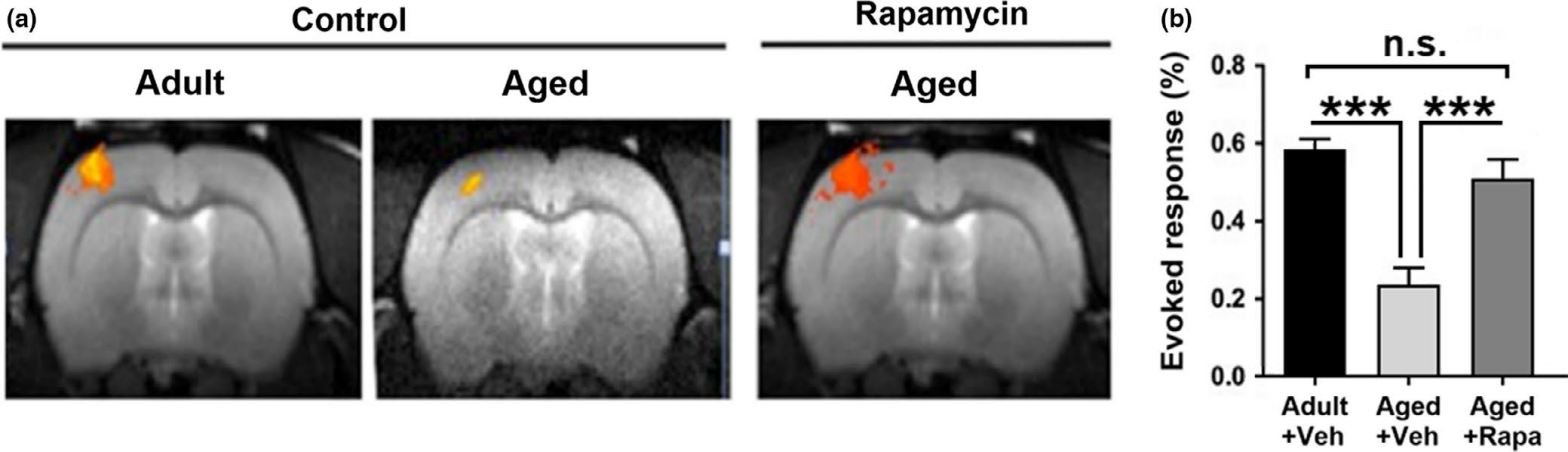
mTOR drives impaired neuronal network activation during aging in rats. (a) Representative fMRI activation in the somatosensory cortex and (b) quantitative analysis demonstrates the response to forepaw stimulation is decreased with age (*** indicates *p* < .001 via *t* test). fMRI activation, however, is preserved in aged rats treated with rapamycin (***, *p* < .001 compared with age‐matched controls). The restoration of fMRI activation in response to forepaw stimulation by mTOR attenuation was complete since the magnitude of the evoked response in the rapamycin‐treated aged group was indistinguishable from that of adult rats. Evoked responses are shown as mean percent increase over baseline cerebral blood flow ± *SEM*

Optimal brain function depends on regulation of CBF in response to neuronal activity, through a complex mechanism known as neurovascular coupling (NVC). Decreased NVC occurs during aging in both humans (Fabiani et al., 2014) and rodents (Toth et al., 2014). Functional MRI (fMRI) measures the hemodynamic response of NVC in response to a defined stimulus as an indicator of neuronal activation. Since decreased neuronal activation and impaired cognitive performance during aging have been linked in humans (Cabeza et al., 1997), we used fMRI to determine overall neuronal activity and neurovascular coupling in our rat model of advanced age measured as the hemodynamic response associated with neuronal activation in response to somatosensory (forepaw) stimulation. We found that evoked fMRI response to somatosensory stimulation was blunted in aged rats as compared to adult animals. Chronic mTOR attenuation by rapamycin, however, restored the fMRI response in aged rats to levels indistinguishable from those of adult animals (Figure 2). These results indicate that mTOR attenuation can restore profound deficits in neurovascular coupling responses in aged rats, suggesting that deficient neuronal network activation and/or impaired functional hyperemia during aging are mediated by mTOR.

### 2.3 mTOR contributes to decreased presynaptic density with age

**Figure 3:**
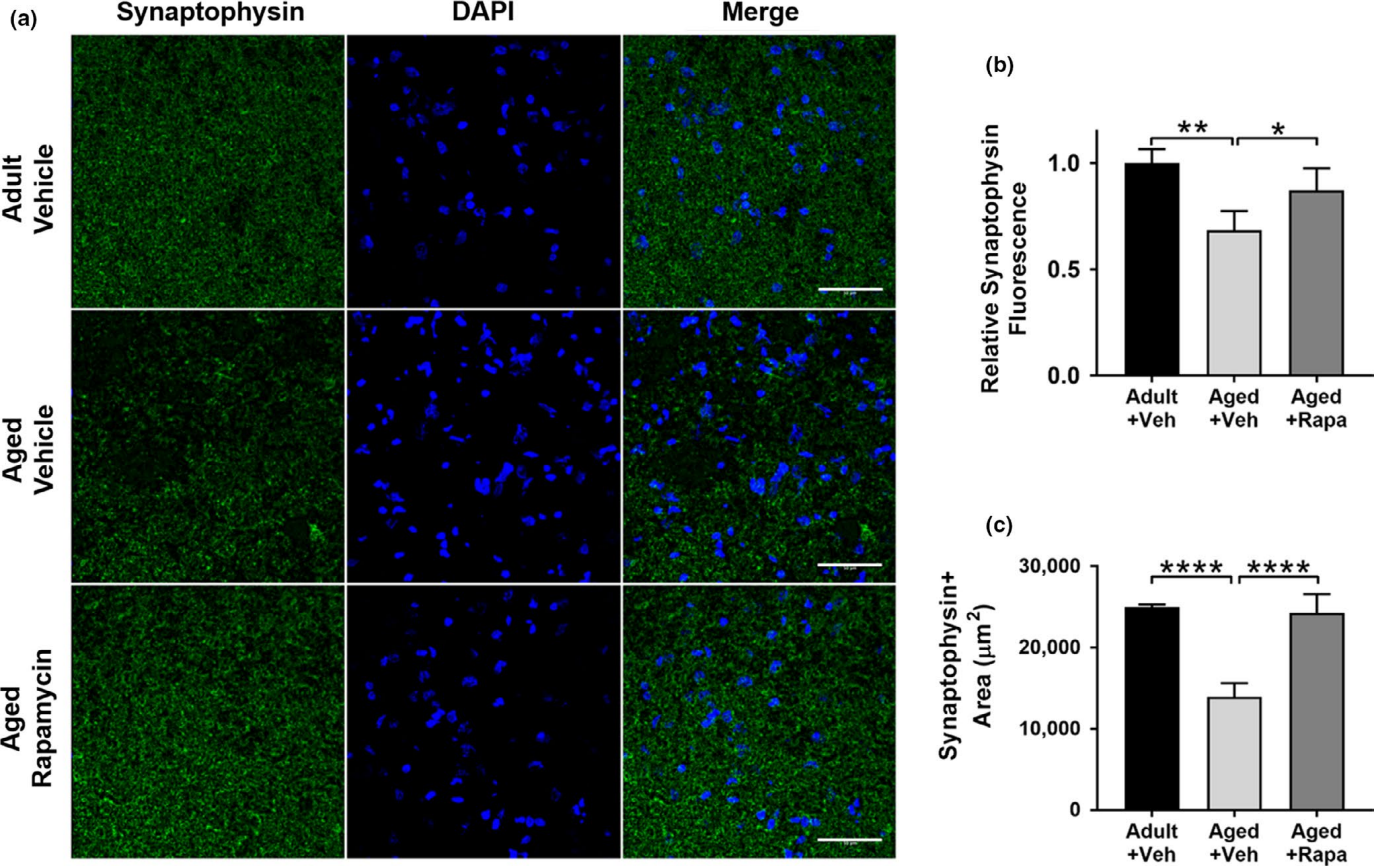
mTOR‐dependent deterioration of presynaptic density during aging. (a) Representative images of synaptophysin immunofluorescent reactivity in hippocampal CA1. (b) Decreased synaptophysin density in aged as compared to adult rats (**, q(9)=7.25, *p* = .002) was significantly ameliorated by chronic mTOR attenuation using rapamycin in the aged + rapa treatment group (*q(9)= 4.31, *p* = .03 vs. aged + vehicle). Synaptophysin density was restored to levels not significantly different from those of adult rats (q(9)=2.93, *p* = .15) in the aged + rapa treatment group. Tukey's *post hoc* tests were applied to a significant omnibus one‐way ANOVA, *F*(2,9)=13.29, *p* = .002). Data are mean ± *SEM* of *n* = 4. (c) Aged rats have fewer synapses as shown by decreased synaptophysin reactive area (****q(9)=13.41, *p* < .0001), a difference that was abolished by mTOR attenuation in aged rats treated with rapamycin (q(9)=12.57, *p* < .0001). Tukey's *post hoc* tests were applied to a significant one‐way ANOVA (*F*(2,9)=56.44, *p* < .0001). Data are presented as mean ± *SEM* of *n* = 4

Since the decrease in functional hyperemia during somatosensory stimulation (i.e., neurovascular coupling) is dependent on the integrity of both neuronal and vascular responses, we quantified presynaptic density to provide a measure of neuronal integrity. Decreased presynaptic density is associated with mild cognitive impairment and AD (Scheff et al., 2015) and with cognitive impairment in rodents (Wang et al., 2014). To define whether changes in synaptic integrity occur during normative aging in rats and understand the role of mTOR, we measured presynaptic density in rats after completion of training and testing in the Morris water maze. Density (Figure 3a and b) and quantity (Figure 3a and c) of synaptophysin‐positive synaptic boutons in hippocampal CA1 were decreased with advanced age in rats. Both density and quantity of synaptophysin‐positive synaptic elements in aged rats treated with rapamycin to attenuate mTOR, however, were indistinguishable from those of adult animals (Figure 3a–c). Together, these findings indicate that chronic mTOR attenuation curtails an age‐related loss of synaptic boutons in the hippocampus, suggesting that preserved presynaptic integrity by mTOR attenuation may underlie the restoration of hippocampal‐dependent learning and memory and the maintenance of fMRI responses to somatosensory stimulation in aged rats. These data suggest that mTOR dysregulation drives age‐related structural remodeling of the hippocampus during aging in the rat and that mTOR attenuation may block age‐related impairments in hippocampal‐dependent memory through the preservation of presynaptic density.

### 2.4 Attenuation of mTOR restores cerebral blood flow and sensory‐evoked functional hyperemia in aged rats

**Figure 4:**
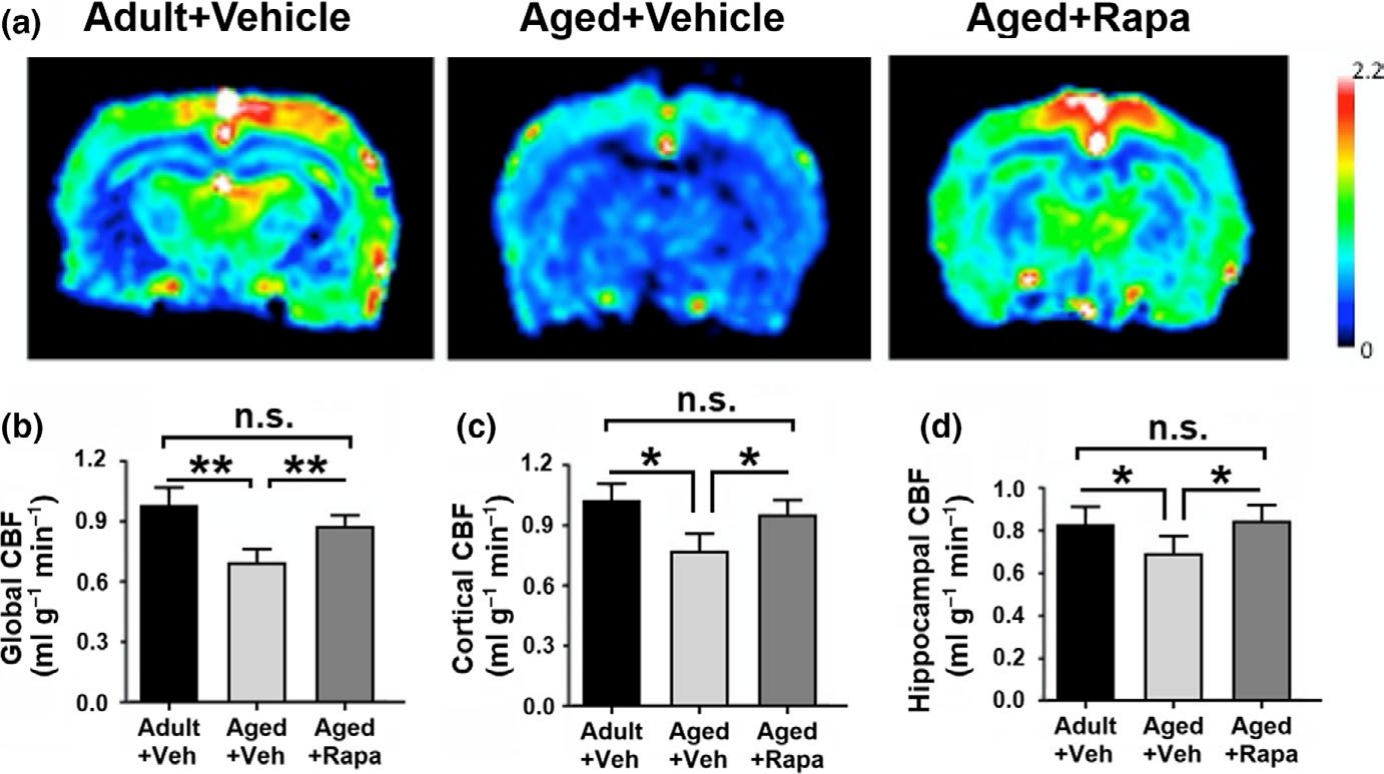
mTOR drives global and regional cerebral hypoperfusion in aging. (a) Representative images of resting CBF obtained by arterial spin labeled MRI. (b) Age‐related cerebral hypoperfusion, evidenced by decreased global CBF (**p < .01) in aged rats, is restored by chronic mTOR attenuation in the aged + rapa group (**p < .01 compared with aged control). (c) Cortical CBF is reduced in aged animals compared with adults (*p < .05), but restored to levels indistinguishable from those of adult rats by chronic mTOR inhibition in the aged + rapa group (*p < .05 compared with aged animals). (d) Reduced hippocampal CBF in aged rats as compared to adults (*p < .05) is abrogated with chronic mTOR attenuation in the aged + rapa group (*p < .05 compared with aged rats) as a result of Student's *t* test

Decreased brain perfusion during normative aging has been established (Melamed, Lavy, Bentin, Cooper, & Rinot, 1980), and we recently showed that mTOR mediates cerebrovascular dysfunction in two different mouse models of AD (Lin et al., 2017, 2013) and in a model of cognitive impairment associated with atherosclerosis (Jahrling et al., 2018). To define the role of cerebrovascular dysfunction in impaired sensory‐evoked functional hyperemia in aged rats (Figure 2), we used arterial spin labeling MRI to measure global and regional resting CBF in adult and aged rat groups (Figure 4). Aged rats had significant impairments in global, cortical, and hippocampal resting CBF as compared to adult animals (Figure 4b‐d), and mTOR attenuation with rapamycin restored global, cortical, and hippocampal resting CBF in aged rats (Figure 4b‐d). These data indicate that mTOR activity drives cerebral hypoperfusion during normative aging.

### 2.5 mTOR attenuation restores cortical microvascular density in aged rats

**Figure 5:**
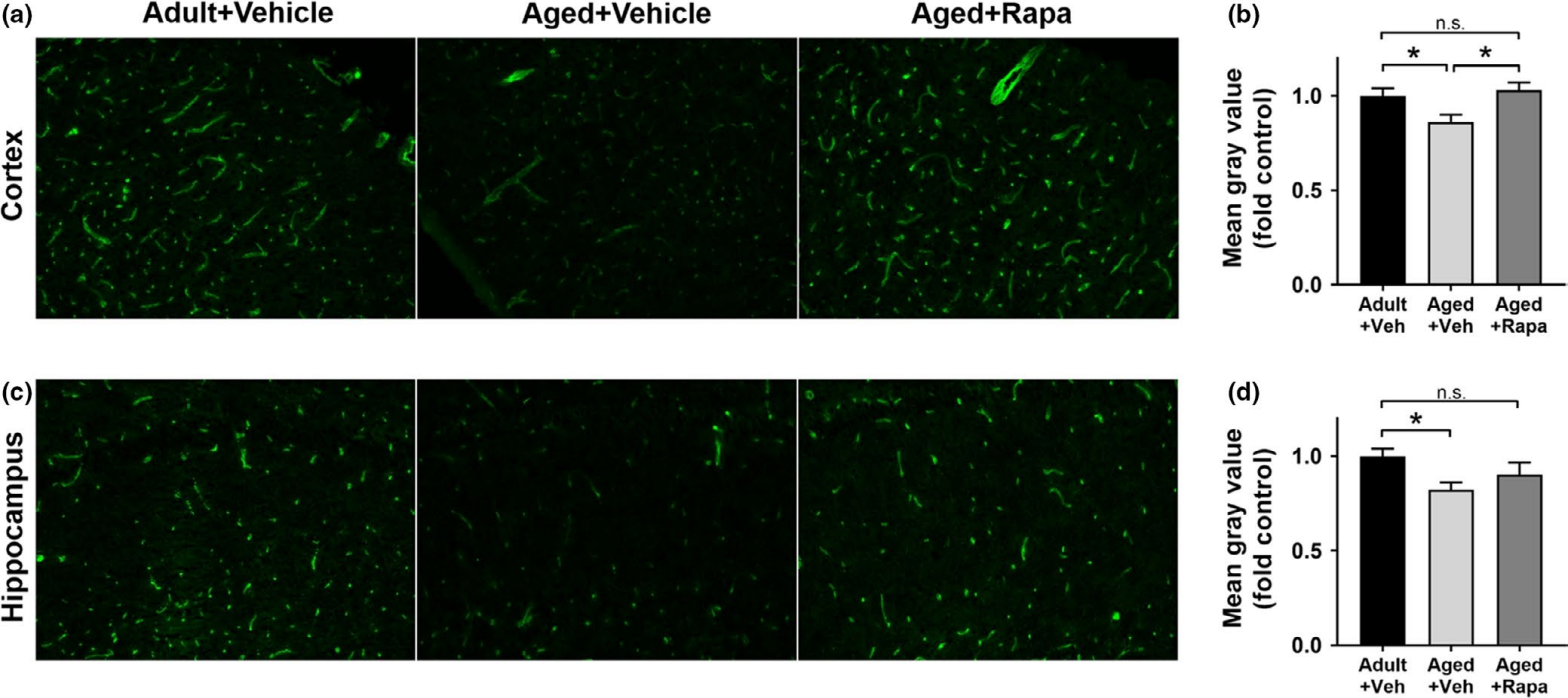
mTOR contributes to age‐related loss of microvascular density in cortex and hippocampus. Representative images of (a) cortical and (b) hippocampal microvasculature highlighted with Alexa488‐tomato lectin labeling of endothelial cells. Quantitative analyses demonstrate decreased microvascular density in (c) cortex of aged rats compared with adult rats (*q(54)=3.48, *p* = .045), which is negated by chronic mTOR attenuation in the aged + rapa group (compared with aged, *q(54)=4.12, *p* = .014); and in (d) hippocampus of aged rats compared with adults (*q(27)=4.19, *p* = .017), which is attenuated with chronic mTOR inhibition in the aged + rapa group (vs. adult, q(27)=1.99, *p* = .35). Data from cortex (a‐b) are from 3 independent fields in 6–7 rats per group for a total *n* = 18–21 in the analysis; data from hippocampus (c‐d) are from 2 independent fields in 4–6 animals per group for a total of *n* = 8–12 in the analysis. Data are displayed as mean ± *SEM* normalized to the adult control group for all studies

Because impaired CBF and blunted functional hyperemia responses could arise from decreased cerebral microvascular density, we assessed cortical and hippocampal microvascular density directly using immunofluorescence in tissue to label microvascular endothelial cells in combination with confocal microscopy and quantitative measures of endothelial cell reactivity on serial sections through parietal cortex and hippocampal CA1. Aged rats showed significantly reduced cortical microvascular density in those brain regions as compared to adult animals (Figure 5a–b). Cortical microvascular density in aged rats treated with rapamycin, however, was indistinguishable from that of adult rats (Figure 5a–b). Similar to cortex, hippocampal microvascular density was significantly decreased in aged rats compared with adults (Figure 5c–d). Attenuation of mTOR, however, restored microvascular density in rapamycin‐treated aged rats to levels indistinguishable from those of adult animals (Figure 5c–d). Taken together, these data indicate that mTOR drives microvascular density loss in cortex and hippocampus during normative aging in rats and implicates mTOR‐dependent microvascular rarefaction in the etiology of decreased CBF and impaired functional hyperemia during aging in rats.

## 3 DISCUSSION

Increased age is the greatest risk factor for AD (Guerreiro & Bras, 2015). Impaired cerebrovascular function during aging (Hamadate et al., 2011;Martin et al., 1991;Wang et al., 2016) is, in turn, a biomarker for increased risk of AD (Zlokovic, 2011) and is one of the earliest detectable changes in the disease pathogenesis (Iturria‐Medina et al., 2016). Consistent with prior reports showing that mTOR inhibition improves learning and memory in aged mice (Halloran et al., 2012;Majumder et al., 2012), our data indicate that chronic mTOR inhibition reduces age‐dependent impairments in spatial learning and memory and that the improved cognitive outcomes are associated with the preservation of synaptic integrity (Figure 3), neuronal network activation (Figure 2), microvascular integrity (Figure 5), and cerebrovascular function (Figure 4) during aging.

Presynaptic synaptophysin expression decreases naturally with nonpathologic aging (Tucsek et al., 2017). Further, lack of functional synaptic protein expression, including synaptophysin, is associated with hippocampal‐dependent memory impairment (Schmitt, Tanimoto, Seeliger, Schaeffel, & Leube, 2009). Consistent with these data, we found that mTOR activity decreased synaptophysin quantity and density (Figure 3) in the hippocampus, suggesting that age‐related synaptic loss may underlie impairments in neuronal network activation (Figure 2) and may contribute to spatial learning and memory deficits (Figure 1) in aged rats. Although mTOR is essential for synaptic function, there is a critical level of mTOR activity that produces optimal synaptic function. For instance, near complete inhibition of mTOR activation during LTP with pharmacogenetics (Stoica et al., 2011) and hyperactivation of mTOR arising from functional loss of negative regulators of the kinase (Lugo et al., 2014) are both detrimental to neuronal function. In contrast, studies have demonstrated that moderate reduction of mTOR activity by ~25%–30% consistently improves aspects of brain function in models of aging (Halloran et al., 2012) and of age‐associated neurological disease (Jahrling et al., 2018; Lin et al., 2017, 2013; Van Skike et al., 2018). Additionally, the mild effects of mTOR inhibition on neuronal function may be a consequence of the relatively poor blood–brain barrier permeability of these compounds (~12,500 fold lower in brain relative to blood, as reported in the methods section). These data suggest the relationship between mTOR activity and cognitive function follows an inverted U‐shaped dose‐effect curve, where very low and very high levels of mTOR activity are deleterious, and enhancement of outcomes (or restoration of disease‐induced deficits) is observed at moderate levels of mTOR, with maximum functional performance occurring at levels of mTOR activity that are reduced by 25%–30% relative to control baseline.

The regulation of CBF during neuronal network activation is a tightly coupled process that depends on the complex interaction between neurons, astrocytes, and vasculature in a process known as neurovascular coupling (Tarantini et al., 2018). Neurovascular coupling reflects both the neuronal activity and the corresponding hemodynamic response that ensures delivery of critical metabolic substrates, largely glucose and oxygen, to active neuronal networks (Tarantini et al., 2018). Chronic mTOR inhibition prevented age‐related declines in global and regional resting CBF, an early biomarker of brain aging in humans (Martin et al., 1991;Schultz et al., 1999). Age‐related cerebral hypoperfusion could be partially explained by microvascular rarefaction, since we found a profound decrease in microvascular density in cortex and hippocampus of aged rats (Figure 5). Microvascular density loss during aging, however, was negated by chronic mTOR attenuation, suggesting that preservation of microvascular density by mTOR attenuation may underlie the protection of CBF in rapamycin‐treated aged rats. These data strongly implicate mTOR as a driver of microvascular rarefaction and dysfunction during aging, and reveal mTOR attenuation as a potentially useful approach to diminish early age‐related CBF loss (Martin et al., 1991; Schultz et al., 1999).

The studies discussed above indicate that mTOR directs aspects of both neuronal and microvascular dysfunction and loss during normative aging in rats. Attenuation of evoked somatosensory fMRI signals in aged rats as compared to adults and their restoration in rapamycin‐treated aged animals likely reflects the mTOR‐mediated loss of both neuronal and vascular components that are necessary for neurovascular coupling. Thus, mTOR‐driven neuronal and/or microvascular dysfunction may have initiated neurovascular uncoupling during normative aging in rats. Future cross‐sectional or longitudinal studies will identify this apical mTOR‐dependent injury.

It has been suggested that decreased cerebral microvascular density during aging (Sonntag, Lynch, Cooney, & Hutchins, 1997) may underlie reduced microvascular plasticity and synaptogenesis in aged rats after environmental enrichment (Black, Polinsky, & Greenough, 1989). Loss of angiogenesis during aging may also contribute to reduced microvascular density and synaptic loss. Alternatively, loss of synaptic integrity with age may subsequently trigger microvascular dysfunction and disintegration, and it is possible that either event may initiate age‐associated cerebrovascular and neuronal dysfunction.

**Figure 6:**
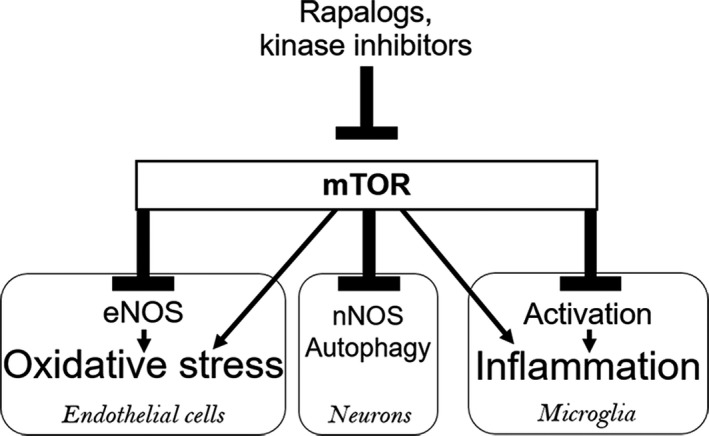
Proposed mTOR‐dependent mechanisms of brain aging. mTOR inhibitors, including rapamycin, rapalogs, and kinase inhibitors, can restore neuronal and cerebrovascular function by blocking specific mTOR‐dependent pathways of brain aging

The beneficial effects of mTOR inhibition we observed in this study may also be related to factors that were not measured in the present study. For instance, mTOR regulates microglial activation states as well as the secretion of proinflammatory cytokines and chemokines (Li et al., 2016). Therefore, some effects may be due to the downregulation of mTOR‐driven neuroinflammatory responses, which increase during aging and negatively impact both neuronal and brain vascular function. Additionally, the central role of oxidative stress in age‐related cerebrovascular dysfunction (Sure et al., 2018) was recently demonstrated in studies showing that the age‐related deterioration of cerebrovascular endothelial cell function and neurovascular coupling responses were reversed by reducing oxidative stress in endothelial cells (Kiss et al., 2019; Tarantini et al., 2019, 2018). Inhibition of mTOR is protective against oxidative stress in vitro in vascular endothelial cells (Zheng et al., 2017), indicating that mTOR may also regulate oxidative stress. While we have not addressed the role of mTOR‐driven neuroinflammation or oxidative stress in our studies, these may represent important mechanisms through which mTOR inhibition ameliorates cerebrovascular dysfunction and cognitive impairment in the aging brain. Figure 6 illustrates the interaction and potential convergence of mTOR‐driven pathways of brain aging.

In summary, we show that mTOR drives cerebrovascular and neuronal dysfunction associated with cognitive decline during normative aging in the rat. In aged rats, chronic mTOR inhibition with rapamycin, an intervention that extends lifespan (Wilkinson et al., 2012), ameliorated mTOR‐dependent decline in learning and memory in aging at least partly through the restoration of cerebrovascular function and synaptic structure. Together with our previous studies, these data indicate that mTOR drives cerebrovascular dysfunction both in normative aging and in age‐associated disease states, including AD and cognitive impairment associated with vascular disease (Halloran et al., 2012; Jahrling et al., 2018; Lin et al., 2017, 2013; Van Skike et al., 2018) and thereby suggests that brain microvascular dysfunction may link aging to an increased risk of AD. Therefore, mTOR attenuation provides a strategy to preserve synaptic and cerebrovascular function during aging and may help to reduce the risk of AD.

Although the safety and tolerability of mTOR inhibition in older adults has generally been demonstrated (Kraig et al., 2018; Mannick et al., 2014), a recent clinical trial in healthy adults over 70 years of age reported relatively mild side effects of daily rapamycin administration for at least 8 weeks, including gastrointestinal issues, facial rash, and stomatitis (Kraig et al., 2018). However, the participants taking rapamycin did not have changes in immune function, insulin sensitivity, insulin secretion, or blood glucose concentration (Kraig et al., 2018). Changes in metabolism and immunological suppression represent significant concerns with chronic mTOR inhibition, but preclinical studies suggest side effects can be managed with dosing regimens that may involve intermittent administration (Arriola Apelo, Pumper, Baar, Cummings, & Lamming, 2016; Dumas & Lamming, 2019). Despite the overwhelming preclinical evidence that rapamycin or other inhibitors of the mTOR pathway may slow the progression of AD and several clinical trials that suggest these medications are well tolerated in older adults, there has not yet been a clinical trial testing rapamycin as a therapeutic to delay or slow disease progression in patients with AD (Kaeberlein & Galvan, 2019). Our findings suggest that mTOR may be a therapeutic target to restore cerebrovascular function during normative aging and that mTOR inhibitors may help decrease the risk of developing age‐associated disorders including vascular‐type dementia and AD (Jahrling et al., 2018; Van Skike et al., 2018). Treatment of age‐related cerebrovascular dysfunction in older adults is expected to prevent further deterioration of brain perfusion, recently identified as a biomarker for the very early (preclinical) stages of AD (Iturria‐Medina et al., 2016), and thus potentially blocking disease initiation and/or progression.

## 4 EXPERIMENTAL PROCEDURES

### 4.1 Animals and treatment conditions

Experiments were performed with approval from the University of Texas Health San Antonio Institutional Animal Care and Use Committee (Animal Welfare Assurance Number A3345‐01), which complies with the Animal Welfare Act, the Guide for the Care and Use of Laboratory Animals, the Public Health Service Policy on Humane Care and Use of Laboratory Animals, the US Government Principles for the Utilization and Care of Vertebrate Animals Used in Testing, Research, and Training, and the ARRIVE (Animal Research: Reporting In Vivo Experiments) guidelines for reporting animal experiments. Sixteen‐month‐ and 34‐month‐old male and female F344BNF1 rats were fed chow containing either microencapsulated rapamycin or vehicle diet containing inactive eudragit capsules. Adult rats were fed vehicle diet for 10 months, beginning at 6 months of age until testing at 16 months. Aged rats were fed either vehicle or rapamycin diets beginning at 19 months of age until testing at 34 months. This formed 3 experimental groups: adult vehicle (*n* = 10), aged vehicle (*n* = 14), and aged + rapamycin (*n* = 15). Aged rats were initially placed on diet containing 42 ppm encapsulated rapamycin for 5 months, but were gradually tapered down to 14 ppm over 2 months due to weight loss and mouth sores observed at the higher dose. Rats remained on 14 ppm diet for the last 8 months of treatment. The average level of rapamycin in uncoagulated blood of aged rats treated with 14 ppm microencapsulated rapamycin in chow was 7.3 ng/ml, which is consistent with studies administering the same diet to C57BL/6 mice (Jahrling et al., 2018). Concentrations found in brain tissue were approximately 12,500 fold lower, at 0.585 pg/mg, which is close to the range of 1–3 pg/mg previously reported in C57BL/6J mice administered the same diet (Lin et al., 2013).

### 4.2 Morris water maze

Rats were trained to navigate to a submerged platform using spatial cues. The pool (150 cm in diameter) was maintained at 23 ± 1°C. Rats were trained for 4 days and received 4 trials per day starting from each of the four quadrants. During each trial, the rat was placed into the maze facing the wall and allowed 60 s to find the platform. If the platform was not located within 60 s, the animal was gently guided to the platform. Rats remained on the platform for 5 s after each trial. When all 4 trials were complete, rats were gently dried and returned to their home cages. A tracking system (Ethovision, Noldus) was used to record the distance traveled to reach the platform and swim speed. Separate two‐way (training day × group) repeated measures ANOVAs were used to analyze distance and swim speed, followed by Tukey's multiple comparison *post hoc* tests among all means.

### 4.3 Functional neuroimaging

The MRI experiments were performed on a horizontal 7T/30 cm magnet (Bruker Biospec) at the Research Imaging Institute of UT Health San Antonio. Anesthesia was induced with 4.0% isoflurane and then maintained at 1.2% isoflurane and air mixture using a face mask. Heart rate (90–130 bpm), respiration rate, and rectal temperature (37 ± 0.5°C) were continuously monitored. A water bath with circulating water at 45–50°C was used to maintain the body temperature. Heart rate and blood oxygen saturation level were recorded using a MouseOx system (STARR Life Science) and maintained within normal physiological ranges.

Quantitative CBF was measured using MRI‐based continuous arterial spin labeling techniques as previously described (Lin, Zhang, Gao, & Watts, 2015). Briefly, a surface coil was placed on top of the head and a labeling coil was placed on the chest, over the heart for continuous arterial spin labeling. The two coils were actively decoupled. Paired, interleaved images were acquired with field of view = 12.8 × 12.8 mm, matrix = 128 × 128, slice thickness = 1 mm, 10 slices, labeling during = 2,100 ms, repetition time = 3,000 ms, and echo time = 20 ms. Continuous arterial spin labeling image analysis employed codes written in Matlab and STIMULATE software (University of Minnesota) to obtain CBF values.

To perform the fMRI experiments, two needle electrodes were inserted under the skin of the right forepaw: one between the first and second digits and the other between the third and fourth digits. These electrodes were then fixed with surgical tape, and the stimulation was confirmed by observing digit twitching. The forepaw was stimulated simultaneously in series at 10 mA, 12 Hz, and 3 ms pulse width. A paradigm of 30 s on and 30 s off was used for forepaw stimulation with five repetitions. Imaging was processed and the relative blood oxygenation level‐dependent (BOLD) changes were calculated using an in‐house Matlab software.

### 4.4 Tissue collection and preparation

Rats were sacrificed by isoflurane overdose, and brains were rapidly removed and divided into two halves along the longitudinal fissure. One half was snap frozen in on dry ice and sectioned into 10 μM slices with a cryostat for immunohistochemical analysis. The cortex from the remaining half was dissected and made into lysates for Western blotting.

### 4.5 Immunofluorescent analysis of microvasculature and synaptic density

Slides were fixed in 4% paraformaldehyde, blocked in 5% bovine serum albumin for 1 hr at room temperature, and incubated with either 488‐conjugated tomato lectin (DyLight488 Lycopersicon Esculentum, Vector Laboratories) or synaptophysin primary antibodies (Millipore Sigma, MAB5258) overnight at 4°C. Slides treated with the synaptophysin primary were washed with PBS, Alexa Fluor 488 secondary was applied for 1 hr, then washed. Coverslips were mounted with Prolong Gold antifade mountant with DAPI (ThermoFisher). A secondary‐only control was included (data not shown) to ensure nonspecific background fluorescence was within acceptable limits. Immunofluorescence was visualized using a Zeiss Axiovert 200 m fluorescent microscope with a 100x/1.3 NA Plan‐Neofluar objective and a Zeiss Axiocam MRm camera.

Tomato lectin immunofluorescence was used to determine the endothelial density of the cortical and hippocampal microvasculature, while synaptophysin immunofluorescence was used to determine presynaptic density in the CA1 of the hippocampus. Three sections of the parietal cortex and three sections of the CA1 were imaged in each rat. Mean gray value of tomato lectin and synaptophysin fluorescence, and synaptophysin‐positive area was measured using ImageJ. Measures from the three sections of each rat were averaged and used for analysis. Separate one‐way ANOVAs followed by Tukey's post hoc comparisons were used to analyze the data.

### 4.6 Measurement of rapamycin using HPLC‐tandem MS

Quantification of rapamycin in blood and brain tissue was performed using HPLC‐tandem MS as previously reported (Jahrling et al., 2018; Lin et al., 2013). The HPLC system consisted of a Shimadzu SCL‐10 A Controller, LC‐10AD pump with a FCV‐10Al mixing chamber, SIL‐10AD autosampler, and an AB Sciex API 3,200 tandem mass spectrometer with turbo ion spray. The analytical column was a Grace Alltima C18 (4.6–150 mm, 5 m) maintained at 60°C during the chromatographic runs using a Shimadzu CTO‐10A column oven.

Rapamycin was quantified in rat brain according to the following protocol. Briefly, 100 mg of calibrator or brain samples were sonicated with 10 ml of 0.5 mg/ml ascomycin (internal standard) and 300 ml of a solution containing 0.1% formic acid and 10 mM ammonium formate dissolved in 95% HPLC grade methanol. After sonication, the samples were vortexed and centrifuged at 15,000 g for 5 min at 23°C. Supernatants were transferred to 1.5 ml microfilterfuge tubes and spun at 15,000 g for 1 min. 40 ml of the final extracts was injected into the LC/MS/MS. The ratio of the peak area of rapamycin to that of the ascomycin standard (response ratio) for each unknown sample was compared against a linear regression of calibrator response ratios to quantify rapamycin.

### 4.7 Experimental design and statistical analysis

Three experimental groups of male and female F344xBN rats were utilized: 16‐month‐old adults treated with vehicle chow (*n* = 10), 34‐month‐old aged rats treated with vehicle chow (*n* = 14), and 34‐month‐old aged rats treated with rapamycin in chow (*n* = 15). For all experiments, one‐way or two‐way ANOVAs were used to analyze differences among the group means on the outcome variables. Tukey's post hoc comparison among all means was performed as indicated to clarify group differences. The data that support the findings of this study are available from the corresponding author upon reasonable request.

## AUTHOR CONTRIBUTIONS

VG, ALL, SNA, and KEF conceived the studies and secured funding; CEV, ALL, and VG designed the experiments; CEV, ALL, SAH, RRB, VYS, JJH, JC, SFH, and MAJ performed or supervised the performance of the experiments; CEV, ALL, SAH, JBJ, JC, and VG analyzed the data; CEV, ALL, SAH, SNA, KEF, MHJ, and VG wrote and/or edited the manuscript.
